# Effects of Acute Ingestion of Native Banana Starch on Glycemic Response Evaluated by Continuous Glucose Monitoring in Obese and Lean Subjects

**DOI:** 10.3390/ijerph120707491

**Published:** 2015-07-06

**Authors:** Guadalupe Jiménez-Domínguez, Jorge L. Ble-Castillo, María A. Aparicio-Trápala, Isela E. Juárez-Rojop, Carlos A. Tovilla-Zárate, Deysi J. Ble-Castillo, Carlos García-Vázquez, Viridiana Olvera-Hernández, Bedelia Pérez-Pimienta, Juan C. Diaz-Zagoya, José D. Mendez

**Affiliations:** 1 Endocrinology Department, General Hospital No. 46, Mexican Institute for Social Security, Villahermosa 86060, Mexico; E-Mail: jimenezg03@hotmail.com; 2 Metabolic Diseases Lab, Research Center, Academic Division of Health Sciences, Juarez Autonomous University of Tabasco, Villahermosa 86150, Mexico; E-Mails: iselajua22@yahoo.com.mx (I.E.J.-R.); gbasecs@hotmail.com (C.G.-V.); viryolvera11@gmail.com (V.O.-H.); zagoya@unam.mx (J.C.D.-Z.); 3 Academic Division of Agricultural Sciences, Juarez Autonomous University of Tabasco, Villahermosa 86280, Mexico; E-Mail: sabina52@hotmail.com; 4 Multidisciplinary Academic Division of Comalcalco, Comalcalco 86650, Mexico; E-Mail: alfonsotovillaz@hotmail.com; 5 Scool of Medicine, Montemorelos University, Montemorelos 67530 Mexico; E-Mail: deysible@hotmail.com; 6 Rodolfo Nieto Padrón Children’s Hospital, Secretaria de Salud, Villahermosa 86150, Mexico; E-Mail: bedelia@live.com.mx; 7 Medical Research Unit on Metabolic Diseases, Medical Specialities Hospital, Centro Médico Nacional Siglo XXI (CMN-SXXI), Mexican Institute for Social Security, Distrito Federal 06703, Mexico; E-Mail: mendezf@unam.mx

**Keywords:** native banana starch, resistant starch, obesity, glycemic response, continuous glucose monitoring

## Abstract

An abnormal glycemic profile, including postprandial glycemia and acute glucose spikes, precedes the onset of overt diabetes in obese subjects. Previous studies have shown the beneficial effects of chronic native banana starch (NBS) supplementation. In this study, we examined the effects of acute ingestion of NBS on glycemic profiles by means of continuous glucose monitoring in obese and lean subjects. In a crossover study, obese and lean subjects consumed beverages containing either 38.3 g of NBS or 38.3 g of digestible corn starch (DCS) twice daily during 4 days. On day 5, a 3-h meal tolerance test (MTT) was performed to evaluate glucose and insulin responses. After 1 week of washout period, treatments were inverted. NBS supplementation reduced the 48-h glycemia AUC in lean, obese, and in the combined group of lean and obese subjects in comparison with DCS. Postprandial glucose and insulin responses at MTT were reduced after NBS in comparison with DCS in all groups. However, no changes were observed in glycemic variability (GV) indexes between groups. In conclusion, acute NBS supplementation improved postprandial glucose and insulin responses in obese and lean subjects during 48 h of everyday life and at MTT. Further research to elucidate the mechanism behind these changes is required.

## 1. Introduction

Worldwide, the combined prevalence of overweight and obesity rose by 27.5% for adults and by 47.1% for children between 1980 and 2013. Mexico is among the countries with the highest prevalence of obesity for both adulthood and childhood [1]. Obesity is one of the major risk factors for Type 2 diabetes (T2DM) and cardiovascular disease (CVD). The critical role of a healthy lifestyle in T2DM prevention has been understood from large-scale intervention studies [2,3]. From these findings, it has been shown that lifestyle changes, including a healthy diet, can reduce the incidence of diabetes in high-risk subjects. It is also known that in addition to postprandial hyperglycemia peaks, glycemic variability (GV), understood as upward and downward acute glucose fluctuations abroad a determined range is associated with oxidative stress damage to the cells [4,5,6,7]. These alterations have been linked with the development of diabetes complications and might also be associated with a reduction in time of diabetes onset in obese subjects. Thus, modifications in diet, which can reduce postprandial glycemia excursions or GV, would result in great benefit in diabetes prevention. Previous studies have demonstrated that dietary fibers are associated with improved glycemic control in healthy subjects and in those with diabetes [8]. In this line, resistant starch (RS) is recognized as a dietary fiber that resists digestion in the human small intestine and that reaches partial or complete fermentation in the colon [9]. The short chain fatty acids (SCFA) produced during this process and the increased incret in secretion have been associated with the beneficial effects of dietary fiber on glucose metabolism [10]. 

Previous studies have demonstrated the beneficial effects of corn resistant starch (Hi-Maize^®^) supplementation on glucose metabolism in animal models and in patients with metabolic syndrome or with T2DM [10,11]. On the other hand, unripe bananas are known to be the non-manufactured food with the highest resistant starch content [12]. Native banana starch (NBS), isolated from the variety *Musa* (*AAA group*) *Dwarf Cavendish* (*F*) is widely produced in Tabasco and exhibits a high RS content. We have shown that NBS supplementation induced a reduction in fasting glycemia and lipids in diabetic rats as well as in those fed with a high-sucrose diet [13,14]. In obese women with diabetes, NBS 24 g/day for 4 weeks improved insulin sensitivity and reduced body weight [15]. In non-diabetic obese women, NBS 30 g/day for 8 weeks induced fasting glycemia reduction and increased insulin sensitivity [16]. 

Interestingly, the recently developed continuous glucose monitoring system (CGMS) provides much more glycemic information, including magnitude, duration, and frequency of blood glucose levels. This tool can be used over several days in patients under free-living conditions, offering more advantages than the simple fasting glycemia or the oral glucose tolerance test (OGTT). In this study, our objective was to determine the effects of acute supplementation of NBS on glycemic profile estimated by 48-h continuous glucose monitoring and during a standardized MTT.

## 2. Experimental Section

### 2.1. Study Subjects

The subjects were recruited from among workers and students of the School of Nutrition of the “Universidad Juarez Autonoma de Tabasco” between January 2013 and December 2013. The experimental protocol was approved by the Ethical Committee of the “Instituto Mexicano del Seguro Social” with number R-2011-2701-50, in compliance with the ethical principles and guidelines for the protection of human subjects under research. The purpose and risks of the study were explained to the participants before they provided written informed consent.

Forty five subjects were screened for obese and lean participants. Anthropometric indexes and basic laboratory examinations were carried out. Participants for the obese group were included if they were healthy persons between 18 and 45 years of age, were obese (World Health Organization [WHO] criterion body mass index [BMI] >30 Kg/m^2^), had a normal fasting glycemia (NFG) with values <100 mg/dL (<5.6 mmol/L), glycated hemoglobin (HbA1c) <6.5% (American Diabetes Association [ADA], 2012), and had maintained stable weight during the three months prior to experimentation. Subjects in the normal-weight groups (lean group) were included if they were between 18 and 45 years of age, had 18.5–24.9 BMI values, had fasting glycemia values < 100 mg/dL (<5.6 mmol/L), had HbA1c values < 6.5%, and had maintained stable weight during the three months prior to experimentation. Subjects not included in this study were those with a previous diagnosis of diabetes, with fasting glycemia > 126 mg/dL (>7.0 mmol/L) or glycated hemoglobin > 6.5% (ADA criteria), with digestive disorders, chronic diseases such as renal or hepatic, being pregnant, under psychiatric treatment, receiving medical or naturist treatment to reduce BW, practicing intense physical activity (>90 min per week), receiving immunosuppressants, or with a history of cigarette smoking or alcoholism. Thirty two participants were eligible for the study, 15 for the obese group and 17 for the lean group. However, only 10 subjects in each group completed the whole protocol. 

### 2.2. Test Products

Native banana starch (NBS) was obtained from unripe (green) bananas (*Musa* [*AAA group*]) *Dwarf Cavendish* (*F*) with a physiological age of 15 weeks obtained from a fruit packing plant located at Km. 43.5 of the Villahermosa-Teapa highway in the Mexican state of Tabasco. The NBS was isolated by means of a previously described procedure [17]. Briefly, after washing, the bananas were peeled, cut into 5–6 cm^3^ pieces, immediately rinsed in citric acid solution, and then macerated at low speed in an industrial blender for 2 min. The homogenate was consecutively sieved through screens (30, 80, and 100-U.S. mesh) and washed with distilled water; it was then centrifuged at 10,000 rpm. The sediment was further purified by washing and centrifugation. The white starch sediment was dried in a convection oven at 40–45 °C, passed through a 100-mesh screen, and stored at room temperature in sealed glass jars. Proximate analysis included the following: 3.38% of moisture content; 1.88% protein; 0.4281% fat, and 0.78% ash (14.003, 14.057, 14.059, and 14.006, AOAC international recommended methods). Water activity (*a_w_*) of NBS was measured using AquaLab equipment and resulted in 0.59. Resistant starch (RS) content measured according to the Goñi *et al.* method was found to be 34% on a dry weight basis [18]. Digestible corn starch (DCS) was purchased from Unilever de México, S. de R.L. de C.V. as Maizena^®^ containing 94.4% rapid digestible starch.

### 2.3. Study Design and Protocol

**Figure 1:**
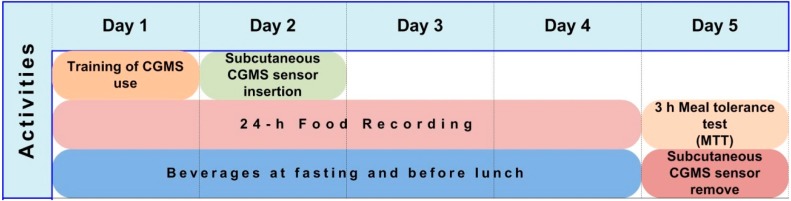
Study Protocol for all participants.

A randomized, crossover, single-blind controlled trial with two 5-day treatment periods and a 7-day washout was conducted. The study protocol for all participants in a 5-day treatment period is shown in Figure 1. On day 1, subjects were trained to use the monitor system and perform the calibrations. On day 2, the CGM system sensor was inserted. From days 1–4 of the treatment period, subjects were under a low-fiber diet, consumed two beverages daily, and were requested to perform daily food recording. On day 5, a 3-h MTT was carried out and the CGMS sensor was removed. After 5 days of supplementation, a 7-day washout period was performed. Supplementation assignments were then reversed for an additional 5 days of follow-up.

### 2.4. Treatments

All of the subjects received low-fiber diets at breakfast and lunch from the Nutrition Department’s restaurant during days 1‒4 of the treatment period. The low-fiber diet consisted of avoiding whole-grain/wholemeal breads and cereals, legumes, nuts, and seeds, and of removing the skins from fruit and vegetables. Subjects were requested to maintain this diet at dinner and to perform daily food recording before they went to bed. Subjects were randomized to receive two beverages daily containing either 38.39 g of NBS or 38.39 g of DCS during 4 days of the treatment period. The NBS beverage contained 38.39 g of NBS, 31.82 g of soy milk, and 240 mL of purified water. The DCS beverage contained 38.39 g of DCS, 31.82 g of soy milk, and 240 mL of purified water. Beverages were consumed in the fasting state (7:00 A.M.–9:00 A.M.) and before lunch (1:00 P.M.–3:00 P.M.). Individuals in the NBS group consumed 26.7 g/day of banana-resistant starch during the first four days of the treatment period. Beverages were prepared and given to the subjects at the restaurant. The doses of NBS used in the present study were based on previous studies from other authors and from our group. Robertson *et al.* administered Hi-Maize^®^ 50 g/day containing 30 g of RS during four weeks to healthy subjects [19] or Hi-Maize^®^ 67 g containing 40 g of RS daily during 12 weeks to patients with T2DM [10]. In previous studies, we employed NBS 30 g/day for eight weeks in obese women without diabetes with good tolerability [16]. Compliance was assessed by counting unopened sachets and by a query regarding the missed servings.

### 2.5. Continuous Glucose Monitoring

All subjects were monitored by the continuous glucose monitoring system (CGMS; Guardian^®^ REAL-Time CGMS; Medtronic, Northridge, CA, USA) for 72 h. On day 1, participants were instructed on how to use the monitoring system and perform, by themselves, at least three calibration readings per day using the capillary blood sampling method (OneTouch^®^ Ultra^®^ 2 from Johnson & Johnson, New Brunswick, NJ, USA). All subjects were instructed to avoid strenuous exercise during the experimental period. The CGM system sensor microelectrode was inserted into subcutaneous (s.c.) tissue in the subjects’ periumbilical region at the clinical site on day 2 and removed on day 5. Criteria for optimal accuracy of the CGM were satisfied [20]. To investigate GV, analysis was limited to data obtained from the intermediate 48-h recording (days 3 and 4) to avoid bias due to insertion and removal of the CGMS sensor. The recorded data were downloaded into a personal computer for analysis of glucose excursions parameters.

### 2.6. Assessment of Glycemic Variability (GV)

Standard deviation (SD) of blood glucose, coefficient of variation (CV), mean amplitude of glycemic excursions (MAGE), and mean absolute glucose (MAG) changes were used to assess GV. The SD was obtained through data management with GraphPad Prism software (La Jolla, CA, USA). MAGE, considered a gold standard of GV, was calculated by measuring the arithmetic mean of the differences between consecutive peaks and nadirs, provided that the differences were >1 SD of the mean glucose value. MAG change takes into account all glycemic variations over time, including those remaining within the physiological range. MAG-5 was obtained by calculating absolute increments and decrements of glucose from peaks to nadirs for 5 min according to the formula MAG = ΔGlucose/ΔTime as described by Hermanides *et al.* [21].

### 2.7. Meal Tolerance Test

A standardized MTT was conducted on day 5 in subjects at fasting state. On arrival at the Investigation Unit, an indwelling catheter was inserted into an antecubital vein in the forearm of the subject for withdrawal of blood samples. Five min after catheter placement, the first blood sample was taken (*t* = 0). Then, the participants were offered their respective beverage and a Kellogg’s^®^ Rice Krispies^®^ bar (Kellogg’s Company, Battle Creek, MI, USA), which had to be consumed at least within 5 min. A 22-g bar of Kellogg’s^®^ Rice Krispies^®^ contains total fat 2 g, total carbohydrates 17 g, protein < 1 g, sodium 105 mg, and dietary fiber, 0. Total calories from the bar were 90 kcal. Considering the nutritional content of the beverages, each subject consumed 391 kcal in this test. Additional blood samples were drawn at 30, 60, 90, 120, 150, and 180 min for glucose and insulin determinations. Subjects remained at the Investigation Unit under observation during the test period and were allowed to read, watch television, or talk. 

### 2.8. Biochemical Measurements

Blood samples were collected by trained personnel and serum samples were immediately frozen and stored at −70 °C until biochemical determination. Glucose, cholesterol, triglycerides, and insulin concentrations were performed using the Architect Clinical Chemistry Autoanalyzer System from Abbott Laboratories (Chicago, IL, USA). Glucose concentration was determined by enzymatic assay and insulin was measured using chemiluminiscent microparticle immunoassay (CMIA). The total coefficient of variation for the glucose and insulin assays were 5% and <7%, respectively. Insulin resistance was estimated according to Homeostasis Model Assessment (HOMA-IR), which was calculated by means of the product of fasting plasma glucose (mmol/L) and insulin (μU/mL) divided by 22.5 [22].

### 2.9. Statistical Analysis

Data are expressed as means ± standard error of the mean (SEM) values, unless otherwise specified. Given the crossover design of the study, endpoint analyses were carried out for completers only. The D’Agostino-Pearson normality test was performed to assess whether the data were consistent with Gaussian distribution. Total area under the curve (AUC) was calculated using the trapezoid method. Two-tailed paired Student *t* test was employed for comparisons between outcomes from the same subjects with different treatments, or with the unpaired Student *t* test for comparisons between obese and lean subjects. Time course data were also analyzed by repeated measures analysis of variance (ANOVA) for comparison. Differences were considered as statistically significant for *p* values <0.05. Data were processed and analysed using GraphPad Prism ver. 6.00 statistical software (GraphPad Software, Inc., La Jolla, CA, USA).

## 3. Results and Discussion

### 3.1. Characteristics of Subjects

From the lean group, seven subjects were excluded, six for technical problems and one for personal reasons. From the obese subjects, three exhibited technical problems and two withdrew informed consent. The main technical problems included the sensor and monitor use and difficulties in performing the recommended daily calibrations. Baseline characteristics at screening of the 20 participants who completed the treatments are displayed in Table 1. The majority of the participants were young women near the age of 30 years. As expected, obese subjects exhibited higher BMI values compared with lean subjects. All of these were classified as type I obese, and 60% exhibited abdominal adiposity according to WHO criteria. All of the participants exhibited fasting normoglycemia according to the ADA, with glycemia values < 100 mg/dL and HbA1c levels < 6.5%. According to the crossover design utilized in this study, each subject received both treatments, NBS and DCS, in a random assignation process.

**Table 1:** Baseline characteristics of the study subjects.

Characteristic	Lean	Obese	*p*
*N* of subjects	10	10	--
Gender (M/F)	1/9	1/9	--
Age (years)	26.4 ± 7.86	31.6 ± 4.97	0.094
BMI (kg/m^2^)	23.27 ± 1.22	30.73 ± 1.07	<0.0001
Glucose (mmol/L)	4.25 ± 0.57	4.60 ± 0.31	0.105
HbA1c (%)	5.18 ± 0.51	5.03 ± 0.58	0.547
Total cholesterol (mmol/L)	4.48 ± 0.92	4.99 ± 0.75	0.192
HDL-Cholesterol (mmol/L)	1.27 ± 0.26	1.23 ± 0.07	0.629
Triglycerides (mmol/L)	1.36 ± 0.84	1.50 ± 0.18	0.620

All values are expressed as mean ± SD. Blood samples were obtained at fasting state. An unpaired Student *t* test was performed. BMI = Body mass index.

### 3.2. Tolerability and Adverse Effects

Mean daily macronutrient intake from 4-day diet diaries did not differ between treatments. Although there was no need to interrupt the experiments, three of ten subjects treated with NBS informed gastrointestinal discomfort manifested as bloating, flatulence, and abdominal cramping. In the DCS group, two of ten participants reported these adverse effects. 

### 3.3. Glycemic Excursions in 48-h CGM

The effects of NBS *vs.* DCS on glycemic characteristics in 48-h CGM are illustrated in Table 2. Mean blood glucose (MBG) and the total glucose AUC-48 h were both significantly reduced after NBS supplementation in comparison with DCS in all of the groups (*p* < 0.0001 for MBG, and *p* < 0.05 for AUC-48-h). No statistical differences were observed between NBS and DCS supplementation on GV indexes, including SD, CV, MAGE, and MAG_5_ changes between groups. 

**Table 2:** Effects of beverages containing native banana starch (NBS) or digestible corn starch (DCS) in glycemic characteristics based on continuous glucose monitoring (CGM).

Characteristic	Lean			Obese			Lean and Obese		
	DCS	NBS	*p*	DCS	NBS	*p*	DCS	NBS	*p*
MBG (mmol/L)	5.83 ± 0.95	5.15 ± 0.71	**<0.0001**	6.28 ± 0.82	5.44 ± 0.63	**<0.0001**	6.06 ± 0.89	5.29 ± 0.67	**<0.0001**
AUC (mmol.h/L)	4.43 ± 0.49	3.97 ± 0.45	**0.0329**	5.85 ± 0.29	5.06 ± 0.20	**0.0121**	5.16 ± 0.32	4.51 ± 0.27	**0.0007**
SD (mmol/L)	1.00 ± 0.44	0.77 ± 0.25	0.1229	1.13 ± 0.39	1.07 ± 0.46	0.7651	1.07 ± 0.41	0.92 ± 0.40	0.2440
CV (%)	16.81 ± 5.88	15.14 ± 4.88	0.3904	17.74 ± 4.60	20.25 ± 9.64	0.5028	17.28 ± 5.16	17.70 ± 7.88	0.8374
MAGE (mmol/L)	2.32 ± 0.67	2.26 ± 0.76	0.8780	2.27 ± 1.07	1.77 ± 0.61	0.2100	2.30 ± 0.87	2.02 ± 0.72	0.2960
MAG_5_ (mmol/L)	0.12 ± 0.04	0.10 ± 0.02	0.2176	0.12 ± 0.03	0.15 ± 0.08	0.3095	0.12 ± 0.04	0.13 ± 0.07	0.7622

Data represent Mean ± SD. AUC = Area under the curve; MBG = Mean blood glucose; SD = Standard deviation; CV = Coefficient of variation; MAGE = Mean amplitude of glycemic excursions, MAG5 = Mean absolute glucose changes for 5 min. A paired Student *t* test was used for comparisons.

When comparing obese and lean subjects, the obese group exhibited a far greater AUC-48-h in comparison with lean subjects in both NBS and DCS supplementation (*p* = 0.034 and *p =* 0.026, respectively). However, no changes were observed in any of the GV indexes between obese and lean groups.

### 3.4. Excursions Exceeding Oral Glucose Tolerance Test (OGTT) Thresholds

Other findings showed that in the lean group, four participants experienced excursions into the impaired glucose tolerance (IGT) range > 7.77 mmol/L (>140 mg/dL) during a period of >2.0 h. In the obese group, two subjects exhibited excursions >11.10 mmol/L (>200 mg/dL) during >1 h and four subjects, excursions of >7.77 mmol/L during a period of >2.0 h. 

### 3.5. Meal Tolerance Test (MTT)

**Figure 2:**
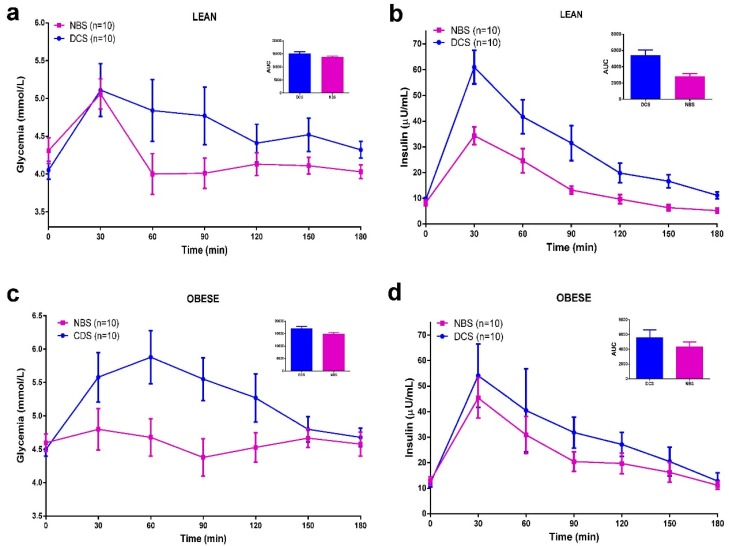

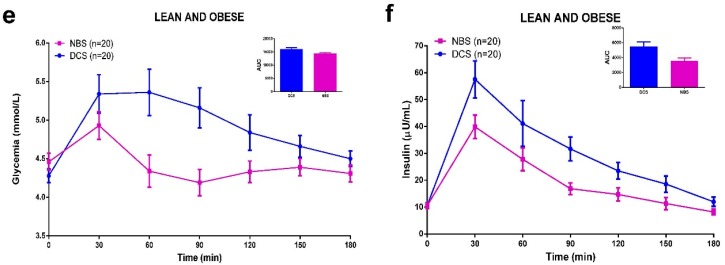
Effects of beverages containing native banana starch (NBS) (red) in comparison with digestible corn starch (DCS) (blue) at the Meal Tolerance Test (MTT) (**a**,**b**) in lean group; (**c**,**d**) in obese group; (**e**,**f**) in lean and obese group. Notes: Beverages were administered 5 min before the standardized food. Areas under the curve (AUC) were compared using a paired Student *t* test or Wilcoxon test. In (**a**) ANB *vs.* DCS *p* = 0.0098; in (**b**) *p* = 0.0018; in (**c**) *p* = 0.0223; in (**d**) *p* = 0.0394; in (**e**) *p* < 0.0001; in (**f**) *p* = 0.0002.

**Figure 3:**
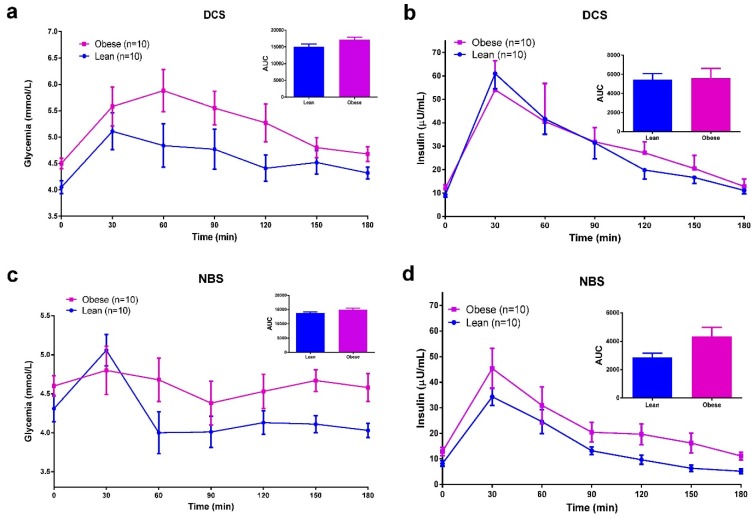
Differences between lean and obese subjects in glycemic and insulin responses during a Meal Tolerance Test (MTT) (**a**,**b**) Beverage containing digestible corn starch (DCS). (**c**,**d**) Beverage containing native banana starch (NBS). Notes: Beverages were administered 5 min before the standardized food. Areas under the curve (AUC) were compared using an unpaired Student *t* test. In (**a**) Obese *vs.* Lean *p* = 0.0853; in (**b**) *p* = 0.8892; in (**c**) *p* = 0.0749; in (**d**) *p* = 0.051.

To better control postprandial glucose and insulin responses a standardized MTT was conducted on day 5. A reduction in glycemic and insulin responses after NBS was observed in all of the studied groups. Total glucose AUC-180 min and total insulin AUC-180 min were both reduced following NBS compared to DCS group (Figure 2). When comparing obese and lean subjects (Figure 3), no differences were appreciated in glucose AUC-180 min or insulin AUC-180 min after DCS. Likewise, there was not significant difference in the glycemic response between groups after NBS. However, insulin response (AUC-180 min) tended to be significantly decreased in lean subjects following NBS (*p* = 0.051). 

### 3.6. Discussion

We showed that acute supplementation of NBS significantly reduces glucose excursions measured by 48-h CGM and also diminished glycemic and insulin responses during a MTT in both lean and obese subjects. However, no changes on GV indexes were observed between groups. In comparison with previous studies, the experimental design of this work is novel in that it employed CGMS to estimate glucose excursions and GV over several days in subjects under conditions similar to those of everyday life. 

All of the participants were included with normal fasting glycemia and normal HbA1c values, however, we could observe that some participants were undergoing excursions into glucose intolerant and/or diabetes range during the 48-h CGM. These results confirm previous observations that dysglycemia may be underestimated by traditional methods such as fasting glycemia and HbA1c, and that CGM could serve as a better tool to detect early alterations [23,24,25]. Likewise, this study also confirmed altered glucose management in obese subjects with normoglycemia and normal HbA1c. Obese subjects exhibited a far greater glycemic burden in comparison with lean subjects regardless of the supplement employed. This disturbance is based on the fact that obese subjects exhibit an early decline in beta cell function, predisposing to an insulin resistance status several years prior to the onset of diabetes.

In both lean and obese subjects, NBS supplementation induced a significant reduction in 48-h glycemic excursions in comparison with DCS. However, in order to better control the postprandial response, an MTT was performed on day 5. In contrast with OGTT, which utilized a pure glucose load, the mixed meal has the advantage of mimicking the “real life” condition. In this test, we observed that NBS significantly reduced glycemic and insulinic postprandial responses in obese and lean subjects. This finding is very important because a recent study in overweight/obese subjects demonstrated that postprandial glycemia is the main contributor to overall 24-h hyperglycemia. Moreover, these authors reported that the contribution is higher when HbA1c values are low, as is the case of many obese subjects with unknown dysglycemia, such as those included in the present study [26].

This effect of NBS in controlling postprandial glycemia under real-life conditions provides great interest with regard to clinical prevention, because it is known that high postprandial glycemia levels occur prior to the deterioration of fasting glucose levels and diabetes onset [27]. Moreover, postprandial hyperglycemia has greater consequences than increased fasting glycemia. In a previous report, increased mortality was observed in subjects with abnormal 2-h postprandial glycemia, but not in those with increased fasting glycemia [28]. In general, it is accepted that hyperglycemic peaks and hyperinsulinemia increases the risk for cardiovascular disease (CVD) in patients with prediabetes [29,30]. A reduction in postprandial responses over long periods could be a preventive factor for reducing the onset of diabetes in high-risk subjects and could diminish the incidence of cardiovascular events in individuals with T2DM [31]. The dietary intervention using products manufactured with NBS might be implemented in order to prevent diabetes and improve cardiovascular prognosis.

Despite altered postprandial glucose excursions, no increased GV indices were observed in the obese subjects group and no effect of NBS on GV was detected. Some of the GV index values, such as SD, CV, and MAGE from this group, were similar to those reported in healthy Chinese population measured in 434 individuals 20–60 years old [32]. The lack of alterations in GV indexes could be partially explained by the relatively youth of the subjects and because only 6 of 10 exhibited a moderated abdominal obesity. Indeed, few studies have investigated GV in normoglycemic populations. Ma Chung-Ming *et al.* in 2011 [33] reported increased GV in abdominally obese subjects with normal glucose tolerance (NGT). However, this study was performed in males, and our study included predominantly women.

Diminished glucose and insulin response after NBS could be partially explained by the reduced rate of digestion [12,34]. However, it could also be attributed to increased short chain fatty acids (SCFA) after RS fermentation in the colon. In previous studies, we demonstrated increased SCFA production in rodent intestine after NBS [14]. Colonic SCFA production has been linked with increased secretion of incretin hormones, such as GLP-1 and PYY, from enteroendocrine cells [19,35]. Plasma SCFA have also shown to inhibit adipose tissue lipolysis *in vivo*: thus, RS might modulate insulin sensitivity through alterations in this fatty acid flux [10,11]. In a recent study in humans, Robertson *et al.* [36] suggested that the most important effect of chronic supplementation with Hi-Maize^®^ was due to an improvement of the adipose tissue physiological function. As a secondary effect, increased glucose uptake into skeletal muscle was followed. The authors found that Hi-Maize^®^ 8-week supplementation induced stimulation of the expression of lipoprotein-lipase (LPL), adipose triglyceride lipase (ATGL), and hormone-sensitive lipase (HSL) in adipose tissue. From these observations, it is clear that further mechanistic studies are required to investigate the effects of NBS. 

Some limitations merit consideration. First, we did not perform an OGTT to rule out glycemic alterations such as diabetes and IGT. Second, this study was conducted predominantly in females because in Mexico, diabetes prevalence is higher in women; thus, the results cannot be extrapolated to male population. Third, we matched the supplements only for total carbohydrates. The advantages of our study, however, comprised the use of a reliable CGMS to investigate glycemic profiles under conditions similar to those of everyday life and the crossover design, which permitted reducing inter-subject variability. 

## 4. Conclusions

In this study, we demonstrated that acute supplementation of NBS improved 48-h glycemic excursions and postprandial glucose and insulin responses in obese and lean subjects. However, no differences in glycemic variability indexes were found between subjects and treatments. These results are relevant because NBS supplementation could represent an inexpensive and accessible alternative to contribute to diabetes and cardiovascular disease prevention in high-risk patients.
